# The effect of suboccipital muscle dysfunction on the biomechanics of the upper cervical spine: a study based on finite element analysis

**DOI:** 10.1186/s12891-024-07401-5

**Published:** 2024-05-21

**Authors:** Rui Li, Yang Liu, Yanzhen Zhang, Can Yang, Zhaojie Zhang, Juying Huang

**Affiliations:** 1 Department of Spine, Wangjing Hospital, China Academy of Chinese Medical Sciences, Beijing, 100102 China; 2 Dongfang Hospital, Beijing University of Chinese Medicine, Beijing, 100078 China; 3 School of Biomedical Engineering, Capital Medical University, Beijing, 100069 China; 4 Beijing Key Laboratory of Fundamental Research on Biomechanics in Clinical Application, Capital Medical University, Beijing, 100069 China

**Keywords:** Muscle dysfunction, Finite element analyse, Atlantoaxial disorders, Suboccipital muscle group, Hypertonia, Cervical spondylosis

## Abstract

**Objective:**

Muscle dysfunction caused by repetitive work or strain in the neck region can interfere muscle responses. Muscle dysfunction can be an important factor in causing cervical spondylosis. However, there has been no research on how the biomechanical properties of the upper cervical spine change when the suboccipital muscle group experiences dysfunction. The objective of this study was to investigate the biomechanical evidence for cervical spondylosis by utilizing the finite element (FE) approach, thus and to provide guidance for clinicians performing acupoint therapy.

**Methods:**

By varying the elastic modulus of the suboccipital muscle, the four FE models of C0-C3 motion segments were reconstructed under the conditions of normal muscle function and muscle dysfunction. For the two normal condition FE models, the elastic modulus for suboccipital muscles on both sides of the C0-C3 motion segments was equal and within the normal range In one muscle dysfunction FE model, the elastic modulus on both sides was equal and greater than 37 kPa, which represented muscle hypertonia; in the other, the elastic modulus of the left and right suboccipital muscles was different, indicating muscle imbalance. The biomechanical behavior of the lateral atlantoaxial joint (LAAJ), atlanto-odontoid joint (ADJ), and intervertebral disc (IVD) was analyzed by simulations, which were carried out under the six loadings of flexion, extension, left and right lateral bending, left and right axial rotation.

**Results:**

Under flexion, the maximum stress in LAAJ with muscle imbalance was higher than that with normal muscle and hypertonia, while the maximum stress in IVD in the hypertonic model was higher than that in the normal and imbalance models. The maximum stress in ADJ was the largest under extension among all loadings for all models. Muscle imbalance and hypertonia did not cause overstress and stress distribution abnormalities in ADJ.

**Conclusion:**

Muscle dysfunction increases the stress in LAAJ and in IVD, but it does not affect ADJ.

## Introduction

Neck pain is one of the most common causes of disability worldwide [1]. The mean age of people experiencing the symptom is continuing to fall [2]. Neck pain can be caused by many pathologies, including those of intervertebral discs (IVD), ligaments, facet joints, and neck muscles [3]. Sustained loading on the cervical spine, such as prolonged use of smartphones or computers, can lead to disc degenerative disease and forward head posture (FHP) [4]. Kuligowski T [5] found that the overall incidence of lumbar segment instability was higher with severer disc damage. FHP affects the length–tension relationship in the suboccipital muscles and causes structural and functional changes in the surrounding muscles, which result in suboccipital muscle dysfunction [6]. The spine can become unstable owing to muscle dysfunction [7]. Sung YH [8] suggested that structural and functional changes in the suboccipital muscles caused by abnormal head posture might be related to cervicogenic dizziness. The suboccipital muscle group is a group of small muscles that connect the occipital bone, atlas, and posterior axis, namely obliquus capitis superior (OCS), rectus capitis posterior major (RCPma), rectus capitis posterior minor (RCPmi), and obliquus capitis inferior (OCI). They are located in the deepest layer of the upper cervical spine [9].

Anatomical studies found that the suboccipital muscle group is crucial for the movement and stability of the lateral atlantoaxial joint (LAAJ) [10, 11]. Stretching the head mainly involves the RCPma, OCS and RCPmi. The ipsilateral flexion of the head mainly involves the OCS. The combined action of the RCPma and OCI causes the face to turn to the same side [12]. Correct posture is considered a state of musculoskeletal balance that involves minimizing stress and strain acting on the body. Lasting reading with lowered head, desk work, and bad posture can induce neck muscle spasms, strain, or relaxation, which leads to imbalances of atlanto-axial intervertebral activities and causes intervertebral joint instability [13]. Li et al. [14] found that patients with musculoskeletal disorders caused by bad postures are often accompanied by an elevation and a left-right imbalance of muscle tonus in the suboccipital triangle region. In summary, muscle dysfunction indicates muscle hypertonia and stiffness, loss of muscle endurance and inability to generate and sustain force on cervical joint with accuracy.

Many in vitro studies demonstrated muscle stabilizes spine. Kettler et al. [15] focused on the effect of cervical muscle forces on the stability of upper cervical spine. It was found that muscle dysfunction results in decreased muscle forces [7], thus leads to abnormal stress at C7-T1 intervertebral disc [16]. Chen et al. [17] demonstrated that spinal stability is more significantly affected by muscle dysfunction than by disc degeneration. Previous studies focused on the effect of sternocleidomastoid, splenius capitis and semispinalis capitis and paid less attention to the suboccipital muscle group in upper cervical region.

Finite element (FE) models representing the human musculoskeletal system offer insight into spinal disorder mechanisms. Kong et al. [18] revealed that lumbar muscle dysfunction destabilized the spine and weakened the role of facet joints in transmitting. Bernier et al. [19] showed that abdominal belts improve spine stability with static muscle force. However, the mechanisms of suboccipital muscle’s effect on the cervical spine are still unknown. This study aims to test the mechanical characteristics of the upper cervical spine under the condition of suboccipital muscle dysfunction by FE method, so as to explore the biomechanical rational of cervical diseases. Our hypothesis is that suboccipital muscle dysfunction caused by prolonged improper postures could destruct normal mechanical environment in cervical region and induce neck pain. The study results are helpful for understanding the effect of muscle dysfunction on the upper cervical spine.

## Materials and methods

### Development of normal C0-C3 model

In this study, a fresh normal cervical spine specimen from a 35-year-old male who died in an accident was selected (provided by the Biomechanics Laboratory of Southern Medical University). An axial CT scan of the C0-C3 motion segment was performed using Aquilion 16, a 16-slice spiral CT manufactured by Toshiba Corporation, Japan. The slice interval for the images in DICOM format was 0.6 mm. The Medical Ethics Committee of Hospital pre-approved the study protocol.

The image data were imported into MIMICS 15.0 to build a three-dimensional (3D) vertebral model of C0-C3. Since the surface of the 3D vertebrae model was very rough after this step, the model was imported into Geomagic 2016 for smoothing, which was conducive to following mesh division and the convergence of FE calculation. To ensure realistic geometric characteristics, a 3D model of IVD was established also using Geomagic 2016 between the lower surface of the C2 vertebral body and the upper surface of the C3 vertebral body. According to the anatomical position of each ligament [20], a transverse ligament (TL), a ligamentum flavum (LF), a joint capsule (JC), a supraspinous ligament (SL), an interspinous ligament (IL), an anterior longitudinal ligament (ALL), and a posterior longitudinal ligament (PLL) were added. This study assumed that muscle path was defined as a straight line segment connecting the starting point of the muscle to its insertion point. These single muscle segments can only interact with their connected endpoints [21]. The suboccipital muscle group, including OCS, RCPma, RCPmi and OCI, were constructed in accordance with their anatomical position.

The 3D vertebra, IVD, ligaments and muscle models were imported into Solidworks to generate solid models, which were then imported into Ansys 19.0 for finite element meshing, and the FE models of the C0-C3 motion segments, including C0, C1, C2, C3, IVD, ligaments, and muscles, were constructed,.

There were four joint contact surfaces between C0 and C3 [21]: the joint contacts between C0 and C1, between the odontoid and C1, between C1 and C2 at the JC, and between C2 and C3 zygapophyseal joints, respectively. Due to the restraint of the transverse ligament, the contact between the odontoid and C1 generates an upward force component under an application of external force, resulting in a relative sliding trend between the odontoid process and the atlas [22]. Therefore, the sliding contact mode was adopted for the contact between the odontoid and C1, and the friction coefficient was defined as 0.10 [23]. For the occiput-atlas joint contact, and the contacts between C1 and C2, between the C2-C3 zygapophyseal joints, and between IVD and the vertebral body, the face-to-face contact method were adopted, and the friction coefficient was also defined as 0.10 [23].

### Material properties

**Fig. 1:**
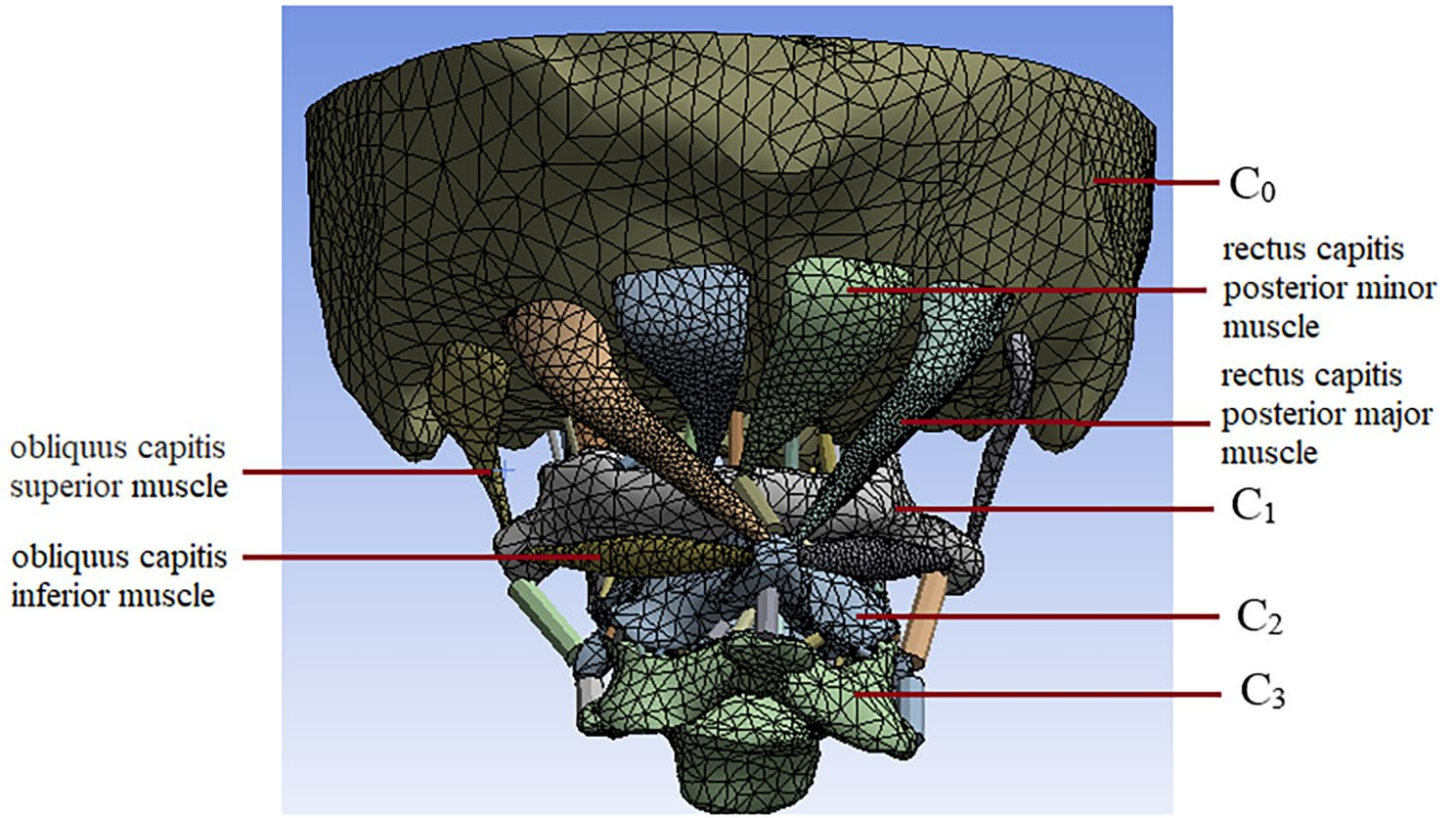
Finite element model of the C0-C3 motion segment

The material properties for the cortical bone, trabecular bone, intervertebral disc, facet joints, and ligament are shown in Table 1. The vertebral bodies, which are composed of a cortical and a cancellous bone, are all simulated by tetrahedral elements and simplified to continuous, homogeneous, and isotropic linear elastic materials [22, 24, 25]. IVD is composed of the annulus fibrosus (AF) and the nucleus pulposus (NP). The AF is divided into tetrahedral shell elements, and the NP is gelatinous with 70–90% water content, which is described by linear viscoelastic material [24]. Ligaments and muscles are fibrous tissues. Ligaments can only withstand tension loadings, and are simulated by linear membrane elements [22, 24–26]. . Muscles can withstand both tension and pressure, so pole elements are used to simulate them. Furthermore, each ligament element and muscle was assigned a cross-sectional area, with values based on the literature [26–28]. Figure 1 shows the normal FE models of C0-C3 motion segment.

**Table 1:** Material properties for various components in the normal FE models

Component	Young’s modulus (MPa)	Poisson’s ratio	Cross-sectional area(mm^2^)
Bone			
cortical bone	12,000	0.29	-
cancellous bone	450	0.29	-
Endplates	500	0.40	
Posterior element	3500	0.29	
AF	8.4	0.49	-
NP	1.3	0.499	-
Articular cartilage	10.4	0.3	-
Ligaments			
TL	20	0.3	46.6
AL	7	0.3	6.8
ALL	30	0.3	6.1
PLL	20	0.3	5.4
JC	20	0.3	13.1
ISL	8	0.3	12.6
LF	9	0.3	50.1
ITL	10	0.3	18.9
Muscles			
OCS	0.2 0.37	0.2 0.2	88 88
RCPma	0.2 0.37	0.2 0.2	168 168
OCI	0.2 0.37	0.2 0.2	195 195
RCPmi	0.2 0.37	0.2 0.2	92 92

AF annulus fibrosus, NP nucleus pulposus, TL transverse ligament, AL alar ligament, ALL anterior longitudinal ligament, PLL posterior longitudinal ligament, JC joint capsule, IL interspinous ligament, LF ligamentum flavum, SL supraspinous ligament, ITL intertransverse ligament, OCS obliquus capitis superior, RCPma rectus capitis posterior major, RCPmi rectus capitis posterior minor, OCI obliquus capitis inferior.

### Simulation of muscle dysfunction

In this study, muscle dysfunction refers to muscle hypertonia and stiffness. The FE models of normal and muscle dysfunctional were simulated by varying the elastic modulus of suboccipital muscles. The elastic modulus of normal muscles ranges from 6.2KPa to 37KPa [29–31]. If the muscle is in a state of hypertonia or stiffness, its elastic modulus is greater than 37KPa. The elastic modulus of suboccipital muscle on both sides of normal C0-C3 motion segments FE model was equal and within the normal range. Muscle dysfunction was considered in the upper cervical spine in the following two cases: (1) the elastic modulus of the left and right suboccipital muscles were equal and greater than 37 kPa, which represented muscle hypertonia; (2) The elastic modulus of the left and right suboccipital muscles was different, which represented muscle imbalance. We constructed two FE models of normal C0-C3 motion segments. One model named FEM1 had the elastic modulus of the suboccipital muscle on the left and right sides set to 20 kPa, and the other model, named FEM2, had the elastic modulus set to 37 kPa. Two FE models with muscle dysfunction were also constructed. One model named FEMDS1 had the elastic modulus of the left and right suboccipital muscles set to 57 kPa, a value beyond the range of normal muscle elastic modulus, and the other model, named FEMDS2, had the elastic modulus of the left and right suboccipital muscles set to 37 kPa and 57 kPa, respectively.

### Boundary conditions and loading patterns

Only appropriate boundary and loading conditions can accurately simulate the entire major movement of the cervical spine, such as flexion, extension, lateral bending, and axial rotation. These settings were as close as possible to those experimental studies that evaluated cervical ranges of motion (ROM). For boundary, the inferior endplates of C3 were fully constrained in all degrees of freedom. All loadings were applied using a rigid surface instead of a single point to prevent any deformation that might occur due to uneven force distribution. A 40 N force was applied to the skull to simulate the head weight, and a moment of 1.0 N·m was applied to simulate the physiological load of the head.

## Result

### Model validation

**Fig. 2:**
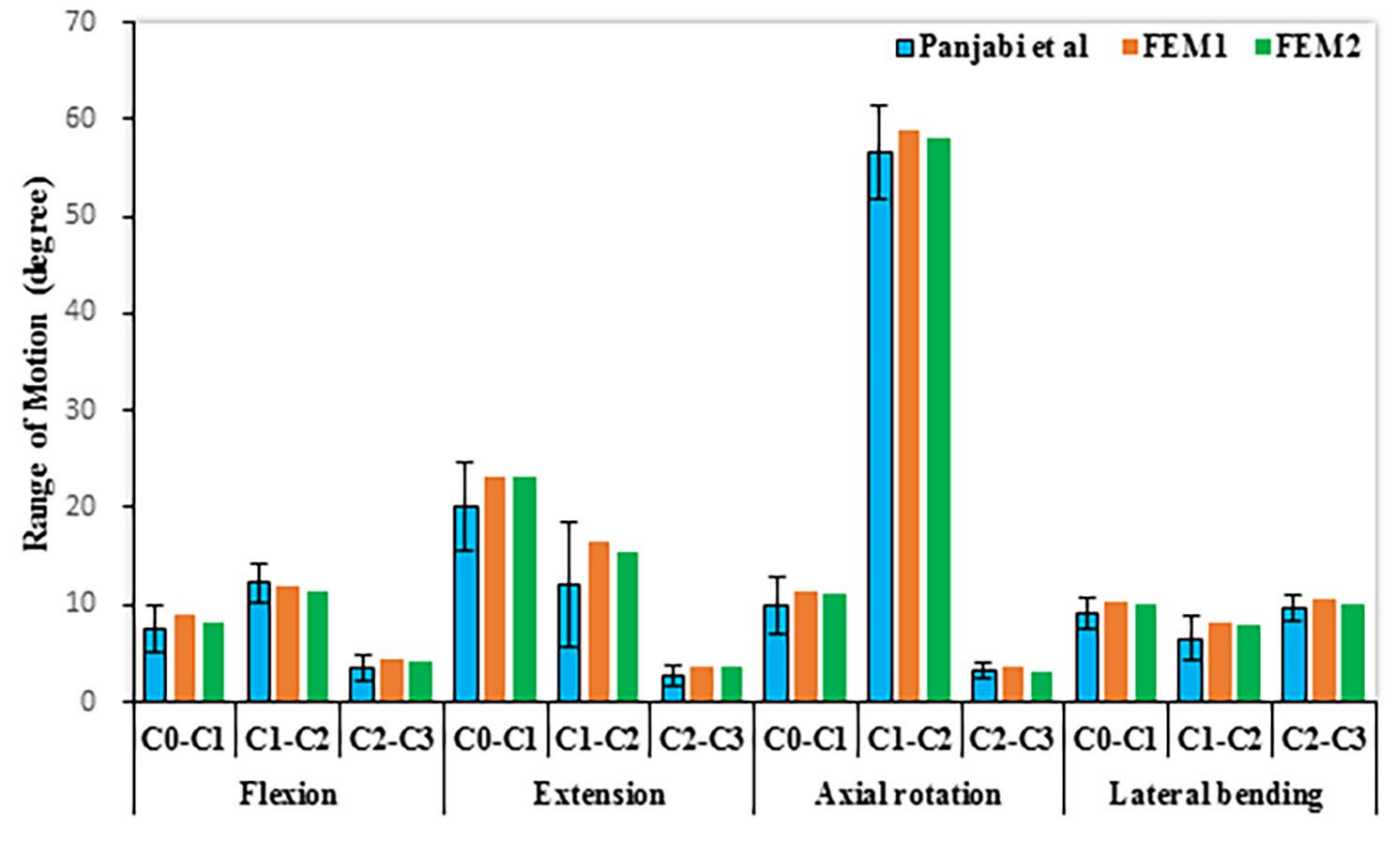
Comparison of ROMs of the finite element models with an in vitro biomechanical study

The normal C0–C3 models established in this study were compared with previously published in vitro experimental results to evaluate its effectiveness. The ROMs of the C0-C3 motion segment are recorded in Fig. 2, which is compared to Panjabi et al.’s study [32]. The values of axial rotation and lateral bending are the sum of the left and right range of motions. Under flexion and extension loading, the maximum ROM occurred at C0-C1, followed by C1-C2 (Fig. 2). The ROMs in flexion and extension, lateral bending and axial rotation were all within the range of the results observed in Panjabi et al.’s study. The C0-C3 normal models, FEM1 and FEM2, have been extensively validated by in vitro studies [32]. Thus, the three-dimensional FE models in this study could effectively reflect the movement of the upper cervical spine.

### Maximum stress of the lateral atlantoaxial joint

**Fig. 3:**
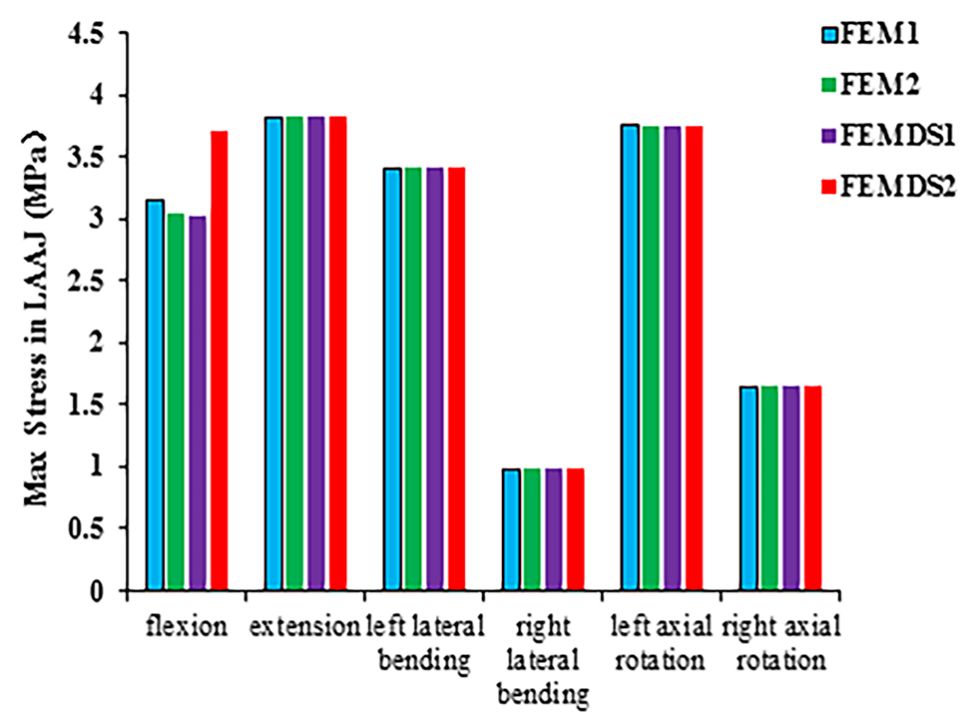
Comparison of the maximum stress of the LAAJ under six loadings across different FE models with varied elastic modulus

Figure 3 shows a comparison of the maximum stresses of LAAJ for the four models under all loadings of flexion, extension, left and right lateral bending, left and right axial rotation. Under flexion, the maximum stress of LAAJ was 3.1515 MPa, 3.0396 MPa, 3.0174 MPa, and 3.7164 MPa for FEM1, FEM2, FEMDS1 and FEMDS1, respectively. The maximum stress of LAAJ was the highest in the FEMSD2 in which the elastic modulus of the left and right suboccipital muscles is different. And the maximum stress gradually decreased as the muscle elastic modulus increased if the elastic modulus of the left and right suboccipital muscle were equal under flexion loading. Under other loadings, the maximum stress of the LAAJ did not change significantly with elastic modulus.

### Maximum stress of C2/3 intervertebral disc

**Fig. 4:**
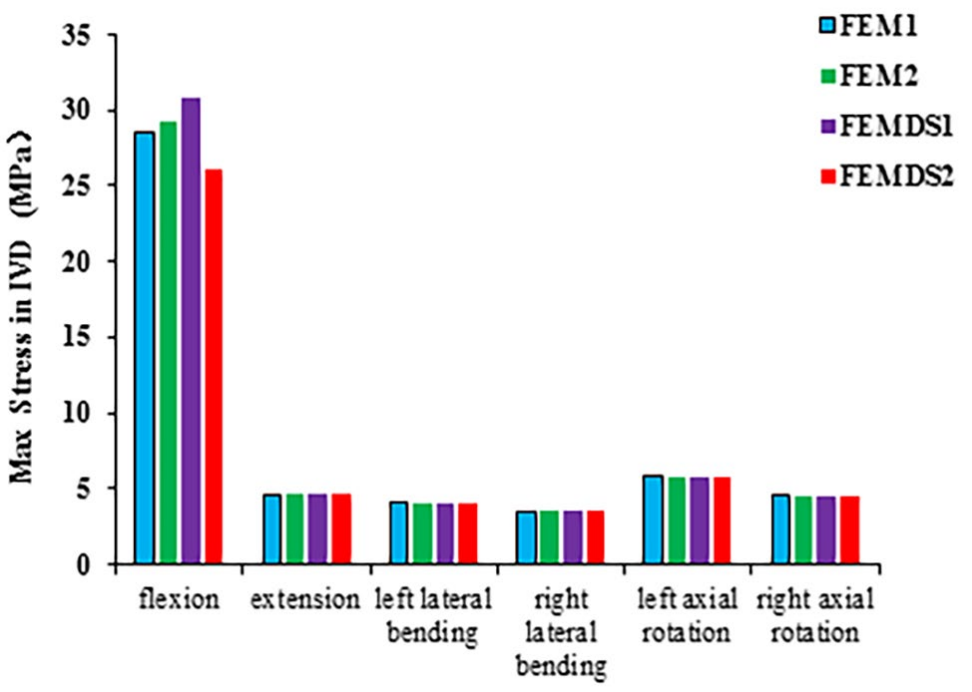
Comparison of the maximum stress in IVD under 6 loadings with across different FE models with varied muscle elastic modulus

Figure 4 shows the comparison of the maximum stress of C2/3 IVD for the four models under all loadings. Compared with other loadings, C2/3 IVDs of the four models were all subject to the largest stress under flexion, with values of 28.565 MPa, 29.189 MPa, 30.816 MPa, and 26.144 MPa for four models FEM1, FEM2, FEMDS1 and FEMDS1, respectively. The value was the smallest in muscle imbalance state, and the highest in muscle hypertonia. For other physiological loadings, with the change in elastic modulus, the maximum stress of C2/3 IVD did not change significantly.

### Maximum stress of the atlanto-odontoid joint

**Fig. 5:**
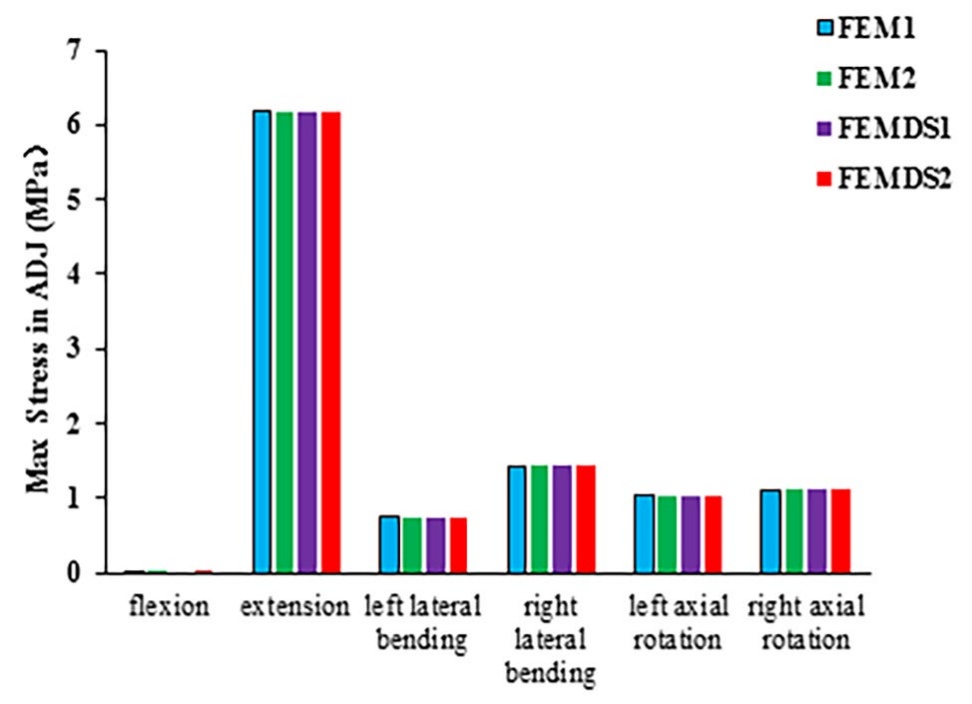
Comparison of the maximum stress in ADJ under 6 loadings across different FE models with varied muscle elastic modulus

Figure 5 shows the comparison of the maximum stress of the ADJ for the four models under all loadings. Compared with other loadings, the stress of ADJ was the largest under extension, which were 6.1778 MPa, 6.1768 MPa, 6.1769 MPa, and 6.1775 MPa for four models FEM1, FEM2, FEMDS1 and FEMDS1, respectively. Under flexion, the stress was all approximately zero for the four models. For all six loadings, the maximum stress value of the ADJ did not change significantly with elastic modulus.

### Stress distribution

**Fig. 6:**
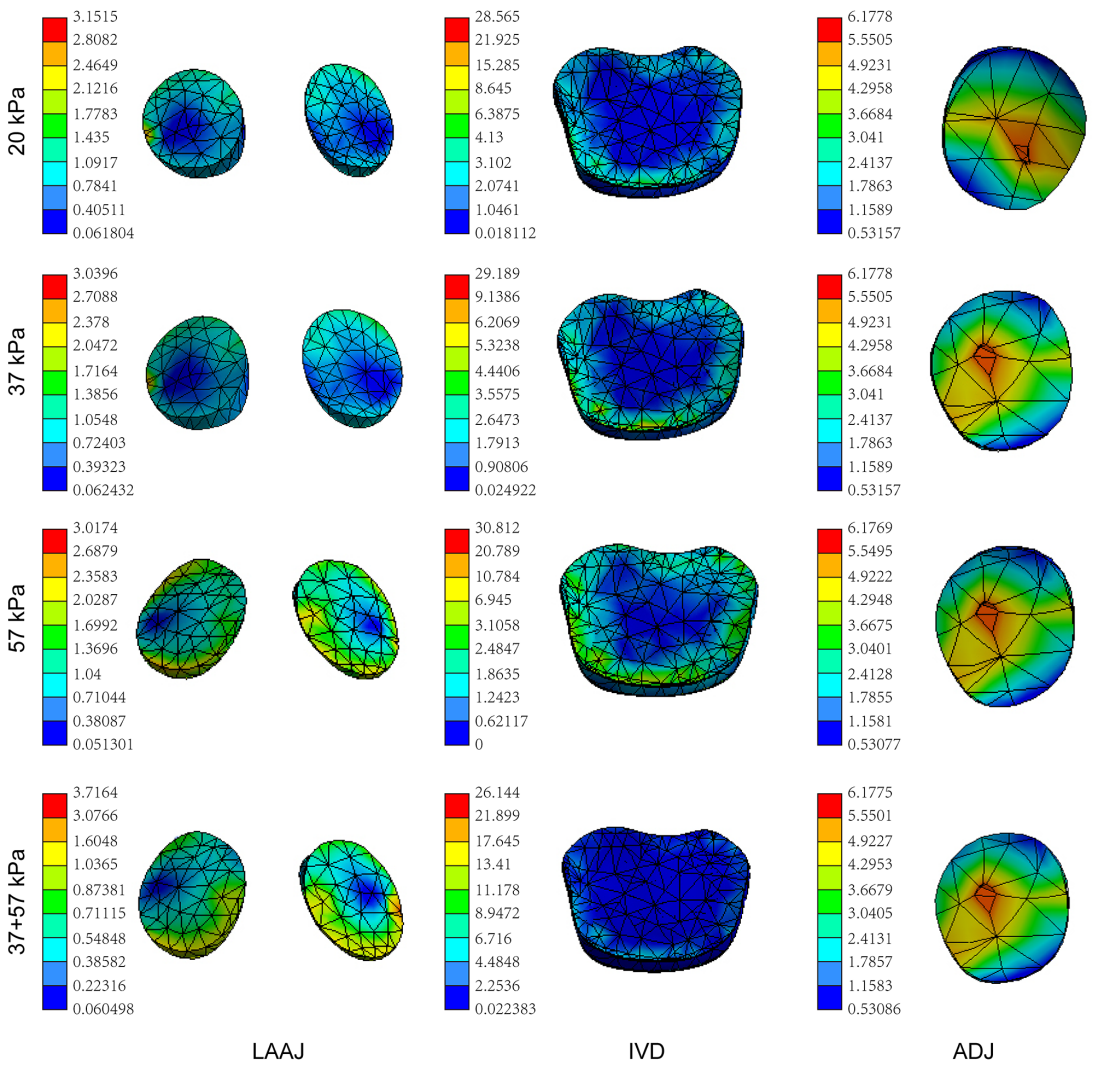
Stress distribution of LAAJ and IVD under flexion and ADJ under extension

Figure 6 shows the stress distribution of LAAJ, IVD, and ADJ of the four models. Under flexion, the stress of LAAJ was concentrated in the anterolateral region of the articular cartilage, and the stress in the central region was small. The stress concentration areas of C2/3 IVD were AF and the endplate; the stress on NP was relatively small. Under extension, the stress of ADJ was concentrated in the central area of the articular cartilage, and the stress in the peripheral was relatively small. There was no significant difference in the stress distribution of the ADJ among the four models.

## Discussion

Muscles are important in maintaining spinal stability by increasing bending stiffness [33, 34]. Muscle dysfunction caused by continuous loading on the cervical spin, such as forward head posture (FHP), can mistake muscle responses [35]. This malalignment of FHP has been suggested to increase stress on the posterior cervical elements, affect the length-tension relationship in the cervical muscles and increase muscular activity level [36, 37]. An increase in neck muscle stiffness can lead to muscle dysfunction and excess strain on the cervical spine [38]. According to some studies [39, 40], micro-damage on suboccipital muscles is induced when excessive weight from FHP accumulates. Uthaikhup et al. [41] reported that structural changes in muscles may be associated with changes in fiber type, functional impairment (reduced strength and endurance), and altered postural and balance control. Functional or structural changes in the suboccipital muscles have been reported to be associated with chronic headache, chronic neck pain, somatic dysfunction, and loss of standing balance [13, 41, 42].

Previous studies [15–119] revealed that lasting improper posture induces musculoskeletal disorders and muscle dysfunction. However, the studies focused on the effect of sternocleidomastoid, splenius capitis and semispinalis capitis and paid less attention to suboccipital muscles in upper cervical region. Early on, our team conducted a few anatomical studies and discovered that the disease’s anatomical basis included [10]: (1) the numerous and complex muscles in the upper cervical region, which are likely to be subject unilateral muscle tension and segment instability after fatigue; (2)the narrow space of the occipital nerve in the suboccipital triangle, where the large occipital nerve runs around the OCI. When the C0 shifts or there is OCI, the nerve can be irritated, resulting in cervico-occipital pain. Muscle dysfunction caused by improper posture may occur initially, followed by neck pain and joint dysfunction. In order to test this hypothesis, the tension of the suboccipital deltoid muscles in 132 patients with neck pain was measured and compared with that of normal people from the literature. It was found that almost all patients with neck pain had muscle hypertonia in the suboccipital triangle and unbalanced tension between the left and right suboccipital muscles [14]. However, the reason of muscle dysfunction resulting in neck pain is still unknown. Therefore, we simulated using FE models the unbalanced and hypertonic state of the muscles and analysed the biomechanical properties of the upper cervical spine when the suboccipital muscle group experiences dysfunction.

The results of FE analysis showed that under flexion, the maximum stress of LAAJ in the FEMSD2 with unbalanced suboccipital muscles was significantly higher than that in FEM1, FEM2 and FEMSD1, which indicates that LAAJ may bear more stress than normal states if the muscle tension is unbalanced under flexion, and this increased stress is likely to cause neck pain. Abnormal pressure on the articular surface may induce the production of cyclooxygenase-2, a proinflammatory enzyme responsible for pain and inflammation [43]. It has been reported that the neck pain is related to a cervical musculoskeletal disorder [44, 45] and Occipital headaches stem from the LAAJ [46, 47]. Thus, our study results are consistent with previous studies and further suggest that the abnormal stress of the LAAJ is the cause of neck pain. Moreover, the fixation of LAAJ relies on the capsule ligament structure, and the increased stress may lead to strain in the joint capsule ligament. Chronic stretching of the capsular ligament from prolonged cervical spine flexion can raise the risk of joint capsule laxity [47].

Prolonged cervical flexion is also crucial for the pathogenesis of atlantoaxial joint disorders, which is reflected by the result that both LAAJ stress increase and disc stress abnormalities occur under flexion loading. Prolonged static posture will lead to muscle fatigue, ischemia, protective muscle contraction, joint fixation, and other conditions [48]. Therefore, neck relaxation and proper posture to maintain cervical lordotic curvature are essential to prevent cervical diseases [49].

The C2-C3 IVD experiences a highest stress under flexion, which is higher than the stress under other physiological loadings. The outcome is consistent with the previous finding that flexion loading subjects the disc to the greatest and most damaging stress [50]. The maximum stress of IVD in the muscle hypertonia model was higher than that in the normal and imbalance models. IVD is subject to high stress as a result of the muscle’s increased tension presumably because the muscle dysfunction undermines the spine’s stability [16]. Although the stress on IVD increased under flexion, the muscle imbalance did not exacerbate this situation, as evidenced by the fact that the maximum stress value under flexion is the smallest in the muscle imbalance model. Chen et al. [51] showed a correlation between the degree of disc degeneration and paravertebral muscle disorder (inflammatory factor expression), and our results support and enrich their view that muscle dysfunction may aggravate disc disease by changing disc biomechanics.

The ADJ was the primary stress joint during extension of the cervical spine. The results of our FE models showed that there was no significant difference in the maximum value and distribution of stress between the models under all loadings, which indicated that muscle dysfunction had limited effect on ADJ. The results are consistent with the clinical observation that ADI in patients with neck pain does not increase in size and is usually less than 3 mm [10].

Cheng et al. [7, 16, 17] demonstrated the relationship between muscle and cervical spine stability, focusing on the effect of muscle dysfunction on range of motion, but paid less attention to the mechanical effect of muscle dysfunction. Our study further revealed the influence of neck muscles on the stress of the upper cervical spine structure, and found that muscle dysfunction could cause stress abnormalities of the articular surface and IVD, which may lead to disc destruction and spinal instability. These results are helpful for guiding clinical practice and are beneficial to the targeted treatment and prevention of neck pain. In addition, the FE method is more controllable and cost-effective than in vitro studies.

Our study has a few limitations. The FE analysis is a simplified solution to complex problems without including active muscle forces, which has a certain impact on the simulation results. But the results are also credible to a certain degree. In addition, the models only considered the influence of muscle dysfunction, without considering the effects of other degenerative changes, such as lordosis reduction and bone hyperplasia. Therefore, a more realistic and accurate FE model should be developed. In future study, it is also necessary to improve the simulation accuracy, and use experimental data to optimize model parameters and verify results, so that the reliability and practicability of the research can be improved.

## Conclusion

FE models were used successfully to reveal the biomechanical effect of muscle dysfunction on the cervical spine. The results showed that muscle imbalance and hypertonia can be a source of pathogenesis of cervical diseases, which is mainly associated with the abnormal increase in stress in LAAJ or IVD under flexion due to muscle dysfunction. Muscle dysfunction does not affect ADJ. Early intervention in muscle imbalance or hypertonia by acupuncture and/or massage may prevent them from developing further and damaging the spine.

## Data Availability

The datasets generated and/or analyzed during the current study are not publicly available due the requirements of patent protection but are available from the corresponding author on request.

## Abbreviations

FHP: Forward head posture
FE: Finite element
LAAJ: Lateral atlantoaxial joint
ADJ: Atlanto-odontoid joint
IVD: Intervertebral disc
OCS: Obliquus capitis superior
RCPma: Rectus capitis posterior major
RCPmi: Rectus capitis posterior minor
OCI: Obliquus capitis inferior CT: computed tomography
3D: Three-dimensional
TL: Transverse ligament
LF: Ligamentum flavum
JC: Joint capsule
SL: Supraspinous ligament
IL: Interspinous ligament
ALL: Anterior longitudinal ligament
PLL: Posterior longitudinal ligament
AF: Annulus fibrosus
NP: Nucleus pulposus
VA: Vertebral artery
ADI: Atlanto-dens interval
ROM: Range of motion
OCS: Obliquus capitis superior
RCPma: Rectus capitis posterior major
RCPmi: Rectus capitis posterior minor
OCI: Obliquus capitis inferior
